# Initial analysis of the synergy of programmed cell death-1 (PD-1) inhibitor and concurrent chemoradiotherapy treatment for recurrent/metastatic head and neck squamous cell carcinoma patients

**DOI:** 10.1186/s13014-023-02310-8

**Published:** 2023-07-04

**Authors:** Lu Li, Lu Chen, Lu Yan, Yueqian Guo, Fang Li, Ming Fan, Mei Lan, Xin Lai, Jie Zhou, Yecai Huang, Peng Xu, Jinyi Lang, Mei Feng

**Affiliations:** 1 Department of Radiation Oncology, Sichuan Cancer Hospital and Institute, Sichuan Cancer Center, School of Medicine, University of Electronic Science and Technology of China, Chengdu, China; 2 Affiliated Hospital of North Sichuan Medical College, Nanchong, China; 3 Chengdu Medical College, Chengdu, China; 4 Department of Clinical Oncology, the Third People’s Hospital of Sichuan, Chengdu, China

**Keywords:** Recurrent, Metastatic, Head and neck squamous cell carcinoma, Chemoradiotherapy, PD-1 inhibitors

## Abstract

**Background:**

Programmed cell death-1 (PD-1) inhibitor was proven to be useful for the recurrent/metastatic head and neck squamous carcinoma (R/M HNSCC) patients. Though both PD-1 inhibitor alone and combination with chemotherapy showed some benefit for PFS and OS, the survival outcome was still not satisfactory. Some studies showed the possible benefit for PD-1 inhibitors combination with radiation for head and neck squamous carcinoma, however there was few studies concerned about synergy of concurrent PD-1 inhibitor combination with chemoradiotherapy for R/M HNSCC. So, we aimed to explore the potential effect and toxicity of the concurrent PD-1 inhibitor and chemoradiotherapy for R/M HNSCC.

**Methods:**

We consecutively enrolled the R/M HNSCC patients treated with concurrent PD-1 inhibitor and chemoradiotherapy from August 2018 to April 2022 in Sichuan Cancer hospital. All the patients received the combination of PD-1 inhibitor and chemotherapy, and followed with synergy of concurrent PD-1 inhibitor and chemoradiotherapy, then maintenance PD-1 inhibitor. ORR and DCR was calculated by immune-related Response Evaluation Criteria in Solid Tumors (irRECIST-1.1), and Common terminology criteria for adverse events (CTCAE-4.0) was used to evaluate the toxicity.The Kaplan–Meier method was used to analyze OS and PFS.

**Results:**

40 R/M HNSCC patients were enrolled in our stuty. The median follow up time was 14 months. 22 patients had recurrent disease only, 16 patients had metastatic disease only, and 2 patients had both recurrence and metastasis disease. For the recurrent lesions, 23 patients received a median radiation dose of 64 Gy (range 50–70 Gy). 18 patients received a median dose of 45 Gy (range 30–66 Gy) for metastatic lesions. The median courses of PD-1 inhibitors and chemotherapy were 8 and 5 respectively. After the treatment, the ORR and DCR were 70.0% and 100%. The median OS was 19 months (range 6.3–31.7 months), with 1 and 2-years OS rates of 72.8% and 33.3%. The median PFS was 9 months (range 3.1–14.9 months), with 6 and 12 months PFS rates of 75.5% and 41.4% respectively. The PFS had no statistical significance in PD-L1 negative and positive group (7 vs 12 months, *p* = 0.059). The most common grade 3 or 4 adverse events(AE) were leucopenia (25.0%), neutropenia (17.5%), anemia (10.0%), thrombocytopenia (5.0%), hyponatremia (2.5%), and pneumonia(2.5%). No grade 5 AE was observed.

**Conclusions:**

The synergy of concurrent PD-1 inhibitor treatment with chemoradiotherapy shows promise as a treatment strategy and an acceptable toxicity for the R/M HNSCC patients.

## Abstract (plain language summary)

Recurrent/metastatic head and neck squamous carcinoma (R/M HNSCC) patients face limited treatment choices and poor prognosis. As a new treatment method, immune checkpoint inhibitor plays an important role for the R/M HNSCC patients recently. However, there were still some controversies for the combination of chemoradiotherapy and immunotherapy, such as timing, radiation dose, fractionation, and et al. In our study, the synergy of concurrent chemoradiotherapy with PD-1 inhibitor showed a promising results for the R/M HNSCC patients, with improved objective response rate (ORR) (70.0%, 95% CI 55.8% to 84.2%) and disease control rate (DCR) (100%, 95% CI 100% to 100%). The median progression-free survival (PFS) and overall survival (OS) were prolonged to 9 months (range 3.1–14.9 months) and 19 months (range 6.3–31.7 months) respectively. The toxicity was tolerable during the treatment, the total incidence rate of grade 3 or 4 adverse events were 65%, similar with other study.

## Introduction

Currently, head and neck squamous cell carcinoma (HNSCC) ranked the eighth of the new cases and deaths in a worldwide [1]. Cancer is an often-fatal neoplasm with only a 50% long-term survival rate. Despite of the advanced development of the treatment technology, R/M HNSCC still has an unfavorable prognosis. Under the EXTREME regimen, the median PFS and OS are 5.6 months and 10.1 months, and the ORR is only 36% [2]. As an alternative TPExtreme solution, the cetuximab combined with platinum and docetaxel has a median PFS of 6 months and OS 14.5 months, and ORR is 57% [3]. Immune checkpoint inhibitors are effective and safe in R/M HNSCC [4]. The phase III KEYNOTE-048 trial of pembrolizumab with or without chemotherapy versus EXTREME in R/M HNSCC. In the total population, pembrolizumab with chemotherapy improved the OS versus cetuximab with chemotherapy (13 months vs 10.7 months), but PFS (4.9 months vs 5.2 months) and ORR (36% vs 36%) were similar [5].

Preclinical data indicate that PD-1 inhibitors and radiotherapy have synergistic effects [6–8], and radiotherapy may stimulate the immune response [6] and immunogenic cell death [9], then release tumor-associated antigen (TAA) [10–12] and enhance the immune cell homing to tumors [13, 14], thereby converting immunologically "cold" tumors into "hot" tumors [15]. Furthermore, radiation-induced increased the PD-L1 expression on tumor cells makes more susceptible to sequential PD-1/PD-L1 inhibitor, resulting in a longer survival time and higher response rate [16]. Besides these, radiotherapy may induce an abscopal effect, with tumor shrinkage occurring beyond the irradiated area [17, 18], and may also enhance T cell recruitment to the tumor microenvironment [19], cytokine secretion, antigen presentation [20, 21]. For LA-HNSCC patients, JAVELIN head and neck 100 trial [22, 23]and KEYNOTE-412 [24] study aimed to explore the efficacy and safety for survival outcome in PD-L1 or PD-1 inhibitors combined with chemoradiotherapy, however both trials got the negative results. Keynote 412 study showed there was a favorable trend toward improved EFS in all the population, and improved OS for patients with CPS≧20, however there was still no significant difference. Recently, for R/M HNSCC patients, some studies began to focus on synergy of PD-1 inhibitor and radiation for metastatic lesions. A phase I/II clinical trial showed the effectiveness of durvalumab plus tremelimumab and stereotactic body radiotherapy (SBRT) in patients with oligometastatic HNSCC, and the ORR was 64.3% and median PFS was 7.2 months**.** The best response rates were encouraging and could be due to the addition of SBRT during immunotherapy that served to either stimulate the immune system or annihilate slow responding or immunotherapy resistant lesions [25]. However, another phase II randomized trial found there was no survival improvement with the combination of SBRT (a single lesion, 9 Gy × 3F) and nivolumab for metastatic HNSCC patients [26]. Till now, there was still controversy, and few studies explored the possibility of concurrent PD-1 inhibitor and chemoradiotherapy for the R/M HNSCC patients, so we aimed to design this trial and find the results out.

## Materials and methods

### Patient selection

This study consecutively enrolled 40 R/M HNSCC patients treated with concurrent PD-1 inhibitor and chemoradiotherapy in Sichuan Cancer Hospital from January 2018 to April 2022. The protocol was approved by the ethics committees. All the patients signed the informed consent.

The inclusion criteria were as follows: age of at least 18 years; Karnofsky Performance Status (KPS) ≥ 60; histologically or cytologically confirmed squamous cell carcinoma; the primary sites were the hypopharynx, oropharynx, oral cavity, larynx, and maxillary sinonasal cavity; recurrent or metastatic disease after primary standard treatment (patients who had recurrent diseases within six months after platinum treatment were excluded); at least one tumour lesion measurable; without previous systemic chemotherapy for recurrent or metastatic disease; adequate renal (estimated creatine clearance > 50 mL/min according to the Cockcroft-Gault formula or ≥ 60 mL/min), liver (total serum bilirubin concentration ≤ 1.5×upper limit of normal and aspartate and alanine aminotransferase concentrations ≤ 2.5×upper limit of normal), and bone marrow (absolute neutrophil count ≥ 1.8 × 10^9^ per L, platelet count ≥ 100 × 10^9^ per L, and haemoglobin ≥ 90 g/L) function. Patients were not selected for PD-L1 status.

The key exclusion criteria included previous treatment with an immune checkpoint inhibitor; symptomatic central nervous system metastases; a history of non-infectious pneumonitis that required glucocorticoids, or active autoimmune disease; severe organ dysfunction or not suitable for chemoradiotherapy.

### Treatment methods

All the patients received 2 cycles of cisplatin-based chemotherapy combined with PD-1 inhibitors, then the synergy of PD-1 inhibitors, platinum-based chemotherapy and radiotherapy. The patients received carboplatin (area under the curve 5 mg/ml/min) or cisplatin (100 mg/m^2^) every 3 weeks. Dose modifications of chemotherapy were permitted according to protocol-specified criteria. Patients received a maximum of six cycles of chemotherapy. Drug treatment was halted if the patient developed severe adverse events (SAE).

PD-1 inhibitors was administered via intravenous once every 3 weeks until disease progression, death, or dose limiting toxicities occurred or the patient requested to stop treatment. There were three kinds of PD-1 inhibitors, and the doses were pembrolizumab 200 mg q3w, camrelizumab 200 mg q3w and toripalimab 240 mg q3w.

All patients received image-guided radiation therapy (IGRT) for recurrent or metastatic measurable lesions. For the recurrent lesions, 23 patients received a median radiation dose of 64 Gy (range 50–70 Gy) with conventional fraction. For the patients with oligometastasis, all the metastatic lesions received the median radiation dose of 45 Gy (30–66 Gy). For the patients with multiple metastasis, up to 5 metastatic lesions received radiation, and the median dose was 45 Gy (30–66 Gy). Among them, 11 patients received hypofractionated radiotherapy (3–10GY/f), and 7 metastasis patients received conventional radiotherapy due to the proximity to organ at risks, such as the mediastinal lymph node, hybercentric and central lung metastatic carcinoma). The dose to organs at risk was set according to the requirements of the Radiation Therapy Oncology Group 0225 (RTOG0225).

### Evaluation criteria

The irRECIST 1.1 was used to evaluate the efficacy of the patients. ORR was defined as the proportion of patients with complete response (CR) or partial response (PR), DCR was defined as the proportion of patients with CR or PR or stable disease (SD). Follow-up was scheduled every 3 months for the first year, and every 12 weeks thereafter. The potential toxic effects of the treatments were assessed each week for the initial three months and at three-week intervals thereafter before immunotherapy courses. Adverse events were classified using the CTCAE 4.0 and the likelihood of an adverse event being immune-related was recorded.

### Statistical analysis

SPSS25.0 software (SPSS Inc. Chicago, IL, USA) was used for statistical analysis. The ORR and 95% CIs were calculated using the Clopper-Pearson method. PFS and OS were analyzed using the Kaplan Meier method. Univariate analysis was performed by logistic regression analysis to determine potential variables as predictors of ORR. Univariate analysis was performed by cox proportional hazard regression for PFS. A two-tailed *p* value of < 0.05 was considered significantly.

## Results

### Patient characteristics

From January 2018 to April 2022, a total of 40 patients were enrolled in our study, the primary sites were oral cavity cancer (*n* = 21), larynx cancer (*n* = 8), maxillary sinus tumors (*n* = 3), hypopharynx cancer (*n* = 6), and oropharynx tumors (*n* = 2). The median age was 53 years (range 32–70), 28 males and 12 females. 16 (40.0%) patients had metastatic disease, with 55.0% (*n* = 22) patients having local recurrence, 2 patients had both recurrence and metastasis sites. The metastasis sites included lung (48.1%), liver (22.2%), mediastinal lymph node (11.1%), bone (11.1%) and brain (7.4%). Programmed death-ligand 1 (PD-L1) were tested for all the patients by 22C3 kits. Among them, 20 patients were positive, 20 patients were negative.22 patients were tested for combined positive score (CPS). Among them, the number of CPS < 1, and ≧1 were 9 and 13 patients respectively (Table 1).

**Table 1:** Basic characteristics

Characteristic	Number	%
*Age*		
< 65	20	50.0
≥ 65	20	50.0
*Sex*		
Male	28	70.0
Female	12	30.0
*KPS*		
< 80	20	50.0
≥ 80	20	50.0
*Primary tumour location*		
Oral cavity	21	52.5
Larynx	8	20.0
Maxillary sinusnasal cavity	3	7.5
Hypopharynx	6	15.0
Oropharynx	2	5.0
*Disease status*		
Distant metastasis	16	40
Local recurrence	22	55
Recurrence and metastasis	2	5
*No. of metastasis*		
1–5	10	55.6
> 5	8	44.4
*Sites of metastasis*		
Liver	6	22.2
Lung	13	48.1
Brain	2	7.4
Mediastinal LN	3	11.1
Bone	3	11.1
*PD-L1 status*		
Negative	20	50.0
Positive	20	50.0

*KPS* Karnofsky Performance Status, *LN* Lymph node, *PD-L1* Programmed cell death ligand 1, *PD-1* Programmed cell death 1

### Treatment methods

PD-1 inhibitor included three different drugs, they were pembrolizumab (200 mg, *n* = 13), camrelizumab (200 mg, *n* = 13), or toripalimab (240 mg, *n* = 14). The median courses of PD-1 inhibitor was 8 (range 6–17), with 16 patients stopping anti-PD-1 treatment because of disease progression, one patient discontinued treatment due to immunotherapy-associated pneumonia (Grade1) after four courses of PD-1 inhibitor, and one patient discontinued treatment due to radiation-induced pneumonia (Grade 3). All the patients received platinum-based chemotherapy. The median courses of chemotherapy were 5 (range 4–6). Radiotherapy was employed using an image-guided intensity modulated radiation therapy (IGRT).

**Fig. 1:**
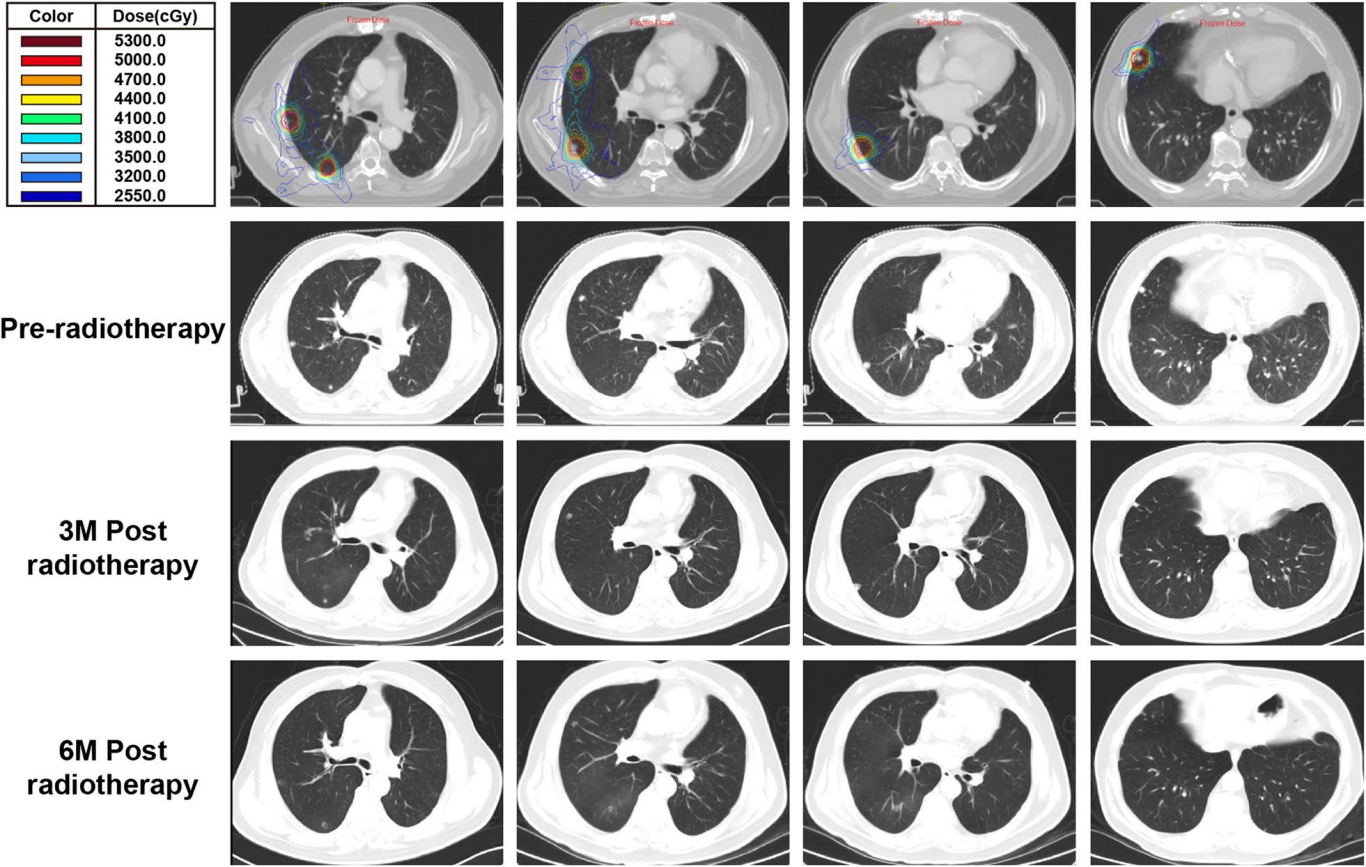
A typical patient for combination PD-1 inhibitor and chemoradiotherapy.The 4 metastatic lesions in right lung received radiation with median dose of 60 Gy (range 50–70 Gy).CT images were showed at pre-radiation, 3 months after the completion of radiation and 6 months after the completion of radiation

24 patients had recurrent diseases, among them, 23 patients received received conventional fractionated radiotherapy with the median dose of 64 Gy (50–70 Gy), and 1 patient received surgery. 18 patients had metastatic lesions (2 patients had both recurrence and metastasis disease). 10 patients were oligometastasis (1–5 lesions), and 8 patients were multiple metastasis (˃5 lesions). The metastatic sites included lung, liver, mediastinal lymph node, bone, and brain. For the patients with oligometastasis, all the metastatic lesions were irradiated with the median dose of 45 Gy (30–66 Gy). For the patients with multiple metastasis, up to 5 metastatic lesions were irradiated with the median dose was 45 Gy (30–66 Gy). Among them, 11 patients received hypofractionated radiotherapy (3-10GY/f), and 7 metastasis patients received conventional radiotherapy due to the proximity to organ at risks, such as the mediastinal lymph node, hybercentric and central lung metastatic carcinoma. Figure 1 showed a typical R/M HNSCC patient.

### Efficacy evaluation

**Fig. 2:**
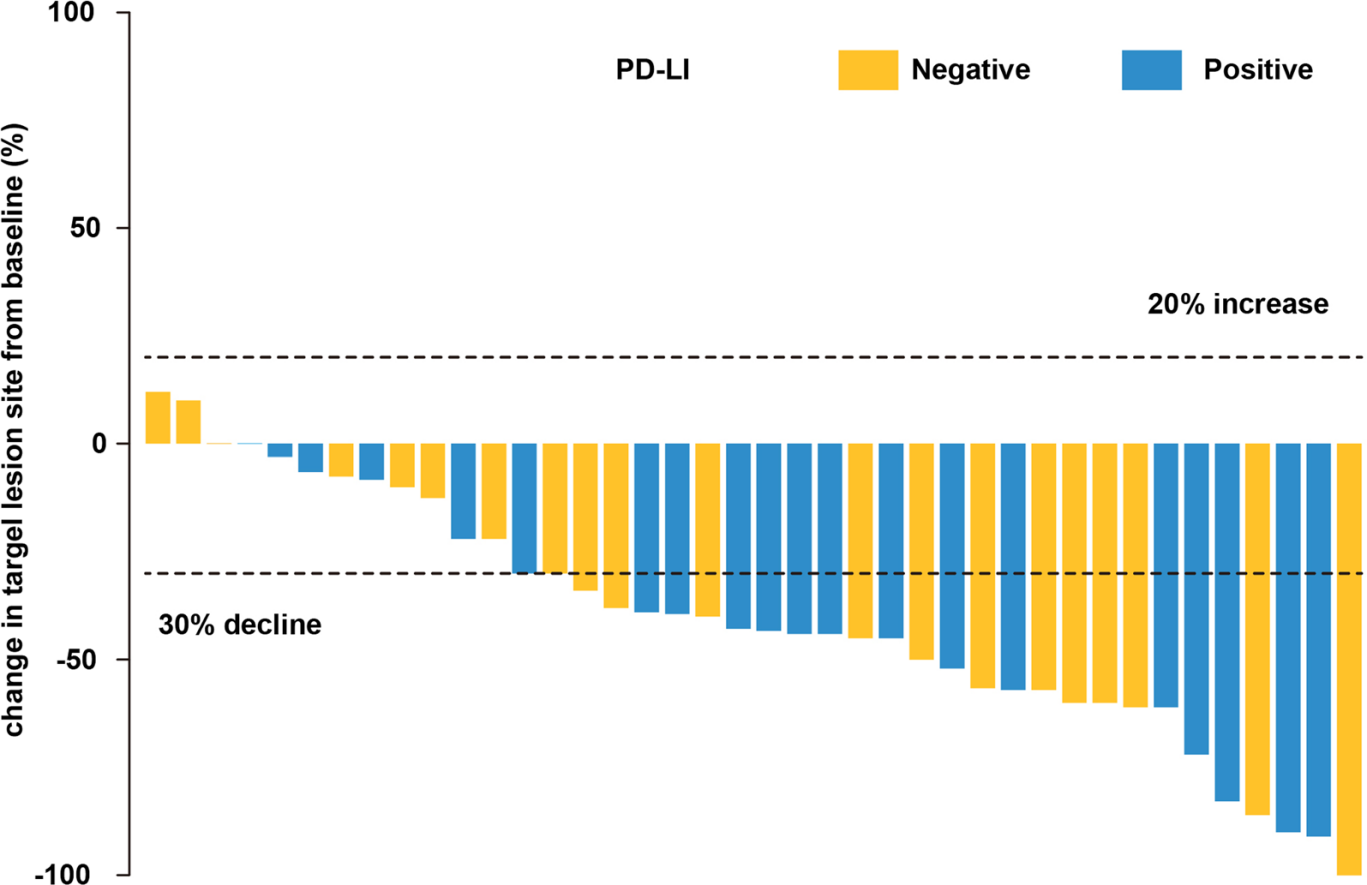
Reductions in target lesion diameters from baseline values per patient

**Fig. 3:**
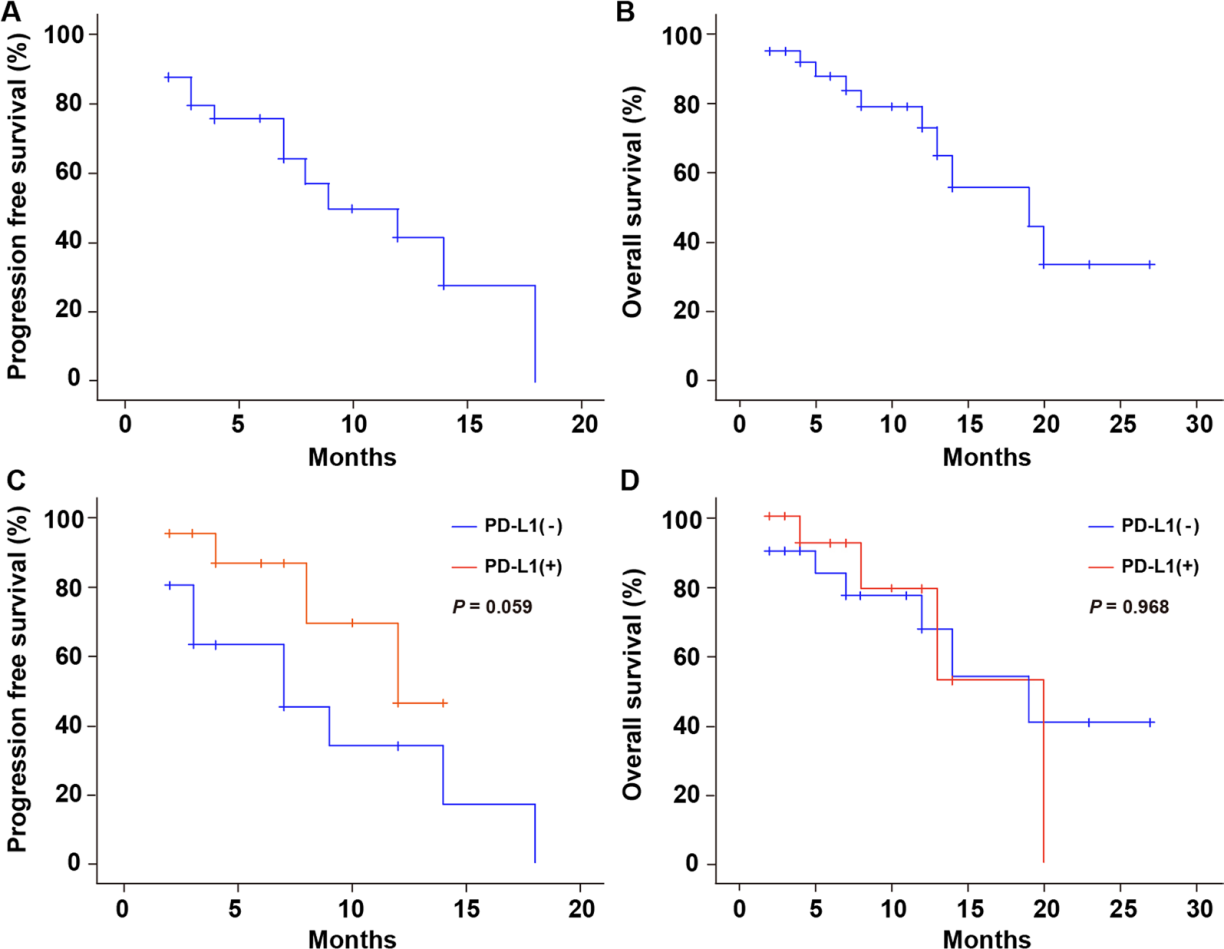
Kaplan–Meier curve displaying limited overall survival in all patients. **A** Progression free survival for all 40 patients evaluated; **B** Overall survival for all 40 patients evaluated; **C** The median PFS was 7 months (range 1.1–12.9 months) and 12 months (range NA) for PD-L1 negative and positive group respectively (*p* = 0.059); **D** OS had no statistical significance in PD-L1 negative and positive group (19 vs 20 months, *p* = 0.968) (Fig. 3D)

The median follow-up was 14 months (range 4–33 months). Among 40 patients, 1 patient (2.5%) had CR, 27 patients (67.5%) had a PR and 12 patients (30.0%) had SD at 3 months after the completion of radiotherapy (Fig. 2). The ORR was 70.0% (95% CI 55.8% to 84.2%), and the DCR was 100% (95% CI 100% to 100%) (Table 2). The median PFS was 9 months (range 3.1–14.9 months), and the median PFS rates at 6 and 12 months was 75.5% (95% CI 62.2% to 88.8%) and 41.4% (95% CI 26.1% to 56.7%), respectively (Fig. 3A). The median OS was 19 months (range 6.3–31.7 months), with the rates at 6,12 and 24 months of 87.7%(95% CI 77.5% to 97.9%), 72.8%(95% CI 59.0% to 86.6%) and 33.3%(95% CI 18.7% to 47.9%), respectively (Fig. 3B).

**Table 2:** Efficacy analysis

Variables	PD-1 inhibitors plus chemoradiotherapy (*N* = 40)
*Overall response at 3 months post radiotherapy*	
Objective response rate	70.0% (95%CI 55.8% to 84.2%)
Disease control rate	100% (95%CI 100% to 100%)
*Progression-free survival (PFS)*	
Median PFS, months	9 (95% CI 3.1 to 14.9)
PFS rate at 6 months	75.5% (95% CI 62.2% to 88.8%)
PFS rate at 12 months	41.4% (95% CI 26.1% to 56.7%)
*Overall survival (OS)*	
Median OS, months	19 (95% CI 6.3 to 31.7)
OS rate at 6 months	87.7% (95% CI 77.5% to 97.9%)
OS rate at 12 months	72.8% (95% CI 59.0% to 86.6%)
OS rate at 24 months	33.3% (95% CI 18.7% to 47.9%)

For univariate analysis (Fig. 3), metastasis number was the only significant factor for PFS (*p* = 0.009), In terms of PD-L1 expression status, there was no significant difference, however it showed the substantial trend for ORR and PFS among patients with PD-L1 positive. The ORR was 75% and 65% (*p* = 0.490), and the median PFS was 7 months and 12 months for PD-L1 negative and positive group respectively (*p* = 0.059)(Table 3, Fig. 3C). The OS had no statistical significance in PD-L1 negative and positive group (19 vs 20 months, *p* = 0.968) (Fig. 3D).

**Table 3:** Univariate analysis resluts

Group	ORR%(95% CI)	*P*-value	PFS months (95% CI)	*P*-value	OS months (95% CI)	*P*-value
*Age(year)*						
< 65	60 (44.8–75.2)	0.168	9 (NA)	0.556	14 (3.0–25.0)	0.180
≥ 65	80 (67.6–92.4)		12 (2.8–21.2)		20 (NA)	
*Sex*						
Male	71.4 (57.4–85.4)	0.763	12 (1.4–22.6)	0.638	19 (9.7–28.3)	0.829
Female	66.7 (52.1–81.3)		9 (7.0–11.0)		14 (3.9–24.1)	
*KPS*						
< 80	65 (50.2–79.8)	0.490	7 (2.0–12.0)	0.124	13 (NA)	0.494
≥ 80	75 (61.6–88.4)		12 (6.8–17.2)		19 (6.3–31.7)	
*Primary tumour location*						
Oral cavity	61.9 (46.9–76.9)		18 (NA)		20 (13.5–28.5)	
Larynx	75 (61.6–88.4)		7 (0–14.1)		NA(NA)	
Maxillary sinusnasal cavity	100.0 (100–100)	0.570	9 (NA)	0.930	14 (NA)	0.219
Hypopharynx	83.3 (71.7–94.9)		7 (0–15.3)		NA (NA)	
Oropharynx	50 (34.5–65.5)		12 (NA)		13 (NA)	
*Disease status*						
Local recurrence	72.7 (58.9–86.5)	0.506	9 (3.7–14.3)	0.260	20 (10.9–29.1)	
Distant metastases	62.5 (47.5–77.5)		7 (0–14.3)		13 (10.2–15.8)	0.432
Recurrence and metastases	100.0(100–100)		NA(NA)		NA(NA)	
*No. of metastasis*						
1–5	85.7(69.5–101.9)	0.237	12 (4.5–19.5)	0.009*	NA(NA)	0.168
> 5	57.1(34.2–80.0)		3 (1.9–4.1)		12(4.5–21.2)	
*PD-L1 status*						
Negative	65 (50.2–79.8)	0.490	7 (1.1–12.9)	0.059	19(10.5–27.5)	0.968
Positive	75 (61.6–88.4)		12 (NA)		20(NA)	

*KPS* Karnofsky Performance Status, *PD-L1* Programmed cell death ligand 1, *NA* Not applicable

^*^ = *P* < 0.05

### Safety analysis

A total of 40 participants were tolerable for toxicities during chemoradiotherapy combined with PD-1 inhibitor. The most common treatment-related adverse events (TRAEs) were leucopenia (80.0%), anemia (77.5%), nausea (77.5%), hyponatremia (70.0%), neutropenia (72.5%), fatigue (70.0%), anorexia (67.5%), constipation (50.0%), vomiting (47.5%), and hyperglycemia (47.5%). 24 patients (62.5%) experienced grade 3 or 4 adverse events that were possibly related to chemotherapy, including leucopenia (25.0%), neutropenia (17.5%), anemia (10.0%), thrombocytopenia (5.0%), and hyponatremia (2.5%). No patient experienced grade 5 TRAEs (Table 4). No SAE or deaths related to the treatment were observed.

**Table 4:** Treatment-related adverse events in all patients (*n* = 40)

Adverse events	Grade1	Grade2	Grade3	Grade4
Anorexia	22(55.0%)	5(12.5%)	0	0
Leucopenia	6(15.0%)	16(40.0%)	9(22.5%)	1(2.5%)
Anemia	12(30.0%)	15(37.5%)	4(10.0%)	0
Nausea	15(37.5%)	16(40.0%)	0	0
Neutropenia	9(22.5%)	13(32.5%)	4(10.0%)	3(7.5%)
Vomit	15(37.5%)	4(10.0%)	0	0
Fatigue	10(25.0%)	18(45.0%)	0	0
Thrombocytopenia	9(22.5%)	3(7.5%)	1(2.5%)	1(2.5%)
Hypoalbuminemia	13(32.5%)	4(10.0%)	0	0
Hypothyroidism	18(45.0%)	0	0	0
ALT increase	4(10.0%)	1(2.5%)	0	0
Constipation	16(40.0%)	4(10.0%)	0	0
Hyponatremia	16(40.0%)	12(30.0%)	0	1(2.5%)
AST increase	3(7.5%)	3(7.5%)	0	0
Fever	9(22.5%)	3(7.5%)	0	0
TSH increase	13(32.5%)	0	0	0
Pneumonia	9(22.5%)	1(2.5%)	1(2.5%)	0
Hyperglycemia	18(45.0%)	1(2.5%)	0	0
UCB increase	3(7.5%)	0	0	0
CB increase	3(7.5%)	0	0	0
Mucositis	4(10.0%)	7(17.5%)	0	0
Hypercreatinine	1(2.5%)	0	0	0
Hypokalaemia	5(12.5%)	1(2.5%)	0	0
Venous thrombosis	5(12.5%)	3(7.5%)	0	0
Rash	1(2.5%)	2(5%)	0	0

All data are presented as No. (%)

*ALT* Alanine aminotransferase, *AST* Aspartate aminotransferase, *TSH* Thyroid-stimulating hormone, *UCB* Unconjugated bilirubin, *CB* Conjugated bilirubin

During the maintenance period of immunotherapy, G1-2 TRAEs were hypothyroidism (45.0%), thyroid stimulating hormone increase (32.5%), rash (7.5%) and immunotherapy-associated pneumonia (2.5%). 7.5% cases (*n* = 3) discontinued PD-1 inhibitor due to adverse events. One patient occured G3 radiation-induced pneumonia, and one patient occured G1 immunotherapy-associated pneumonia. The incidence of G1-4 pneumonia was 27.5% in our study, only one patient had G3 radiation-induced pneumonia (2.5%). Totally 13 patients received the thoracic radiation therapy. The incidence of pneumonia of all grades in the 13 patients was 46.1%, and 5 patients (38.4%) experienced G1-2 pneumonia, 1 patients (7.7%) experienced G3 pneumonia.

## Discussion

In 2020, over 870,000 new cases of HNSCC were diagnosed globally, accounting for 4.5% of all newly diagnosed malignant cancers worldwide [27]. Despite advancements in surgical and chemoradiotherapy procedures, more than half of patients develop a relapse or distant metastases [28]. Cytotoxic chemotherapy and epidermal growth factor receptor (EGFR)-targeting monoclonal antibodies remain the standard treatment option for a recurring disease that is no longer responsive to local therapy or metastatic HNSCC. Based on the results of KEYNOTE-048 [5] and Checkmate-141 [29], the NCCN recommended the PD-1 inhibitor pembrolizumab or nivolumab to the R/M HNSCC alone or combination with chemotherapy. For EXTREME trials, the cetuximab combined with platinum and fluorouracil treatment has an ORR of 36%, the median PFS and OS was 5.6 months and 10.1 months [2]. The KEYNOTE-048 findings showed that pembrolizumab combined with platinum and fluorouracil resulted in the same ORR for all patients, the median PFS and OS was 4.9 months and 13.0 months [5]. Till now, the R/M HNSCC patients face limited treatment options and a poor prognosis.

At present, the phase II/III clinical trials showed there were no significant improvement for the LA-HNSCC patients treated with the chemoradiotherapy combination with immunotherapy. However, for the R/M HNSCC, some basic studies have already showed the potential synergistic effects for the combination of radiotherapy and immunotherapy [6, 30, 31]. Dovedi S.J and et al. evaluated three distinct combination schedules where mice bearing established CT26 tumors received a fractionated radiotherapy with administration of aPD-L1 mAb commencing either on day 1 of the fractionated radiotherapy cycle, day 5 of the cycle or 7 days after completion of radiotherapy. The result showed that only blockade of PD-L1 at the time of radiotherapy delivery can enhance the therapeutic response with sequential therapy [32]. Deng LF and et al. observed that combination of anti-PD-L1 and the ionizing irradiation (IR) of 12 Gy dose on day 14 significantly enhanced the inhibition of TUBO tumor mice growth. Furthermore, the optimal schedule for combining radiotherapy and immune checkpoint inhibitors was still unclear [33]. A retrospective review of 750 solid tumor patients who received immunotherapy (anti-CTLA4 and/or anti-PD-1/anti-PD-L1) and radiotherapy, suggested that overall survival was better for patients who received concurrent immunotherapy and radiotherapy [34]. Other similar phase I/II clinical trial have shown the effectiveness of durvalumab plus tremelimumab and SBRT in HNSCC patients with oligometastatic and the median PFS was 7.2 months [25]. In contrast, a phase II trial of nivolumab versus nivolumab plus SBRT in HNSCC patients with metastatic found no significant difference in ORR (34.5% vs 29.0%, *p* = 5.86) and PFS (1.9 vs 2.6,* p* = 0.79) [26]. In our study, we investigated a new treatment strategy and observed synergy of PD-1 inhibitor and concurrent chemoradiotherapy, and it resulted in 70.0% ORR, and the median PFS and OS was prolonged to 9 and 19 months. These results was favorable. This suggests that the synergy of PD-1 inhibitor and concurrent chemoradiotherapy might be a promising strategy, and it needed to be confirmed in the future.

Preclinical evidence supports that different doses and fractionation schedules would have different pro-immunogenic effects. Hypofraction radiation results in greater stromal and vascular damage and increased apoptosis of tumour cells [35], and creates a tumour microenvironment that is highly enriched with tumour-associated antigens. However, it is unclear about the optimal fractionation and radiation dose. High ablative single doses (> 20 Gy), such as those delivered by SABR/SBRT have been shown to dramatically increase T cell priming, CD8 + T cell infiltration and the induction of tumour regression in breast, lung and melanoma mouse models [36, 37]. One study suggested that a single-dose of 12–18 Gy could attenuate the immunological response of the cell, while three to five daily doses below 12 Gy were more immunogenic [38]. Morisada and et al. [39].observed that hypofractionation of 8 Gy could sustain the infiltration of CD8 T cells while reducing the accumulation of peripheral myeloid-derived suppressor cells (MDSCs). In our study, most of the metastatic lesions were treated with hypofractionated radiation combined with immunotherapy. For some metastatic sites which were next to the blood vessels and some important organs, the conventional fraction was used. Our study got 70.0% ORR and tolerable toxicity.

It can be difficult to determine the range of radiation lesions when multiple metastases were present. Since numerous clinical trials have assessed the use of this combined therapy, most of which have used the single-site [40]. The ST-ICI trial concluded that local radiotherapy to all lesions is more effective than to a single lesion only when combined with PD-1 inhibitor therapy. Patients with PD-1/PD-L1 immune checkpoint inhibitor therapy benefit from local radiotherapy to all known lesions compared to single-lesion radiotherapy regarding PFS (9.2 months vs. 3.0 months) and OS (11.6 months vs. 4.2 months) [41]. The targeting of multiple lesions facilitate TAA priming, as well as bypassing the potential problem of tumor heterogeneity as the broader targeting may allow the recognition of a wider range of TAAs. Brook ED advocate initially attempting comprehensive radiotherapy in combination with ICI in patients with oligometastasis (defined as six or fewer metastases) [40]. For our study, we performed radiotherapy for both oligometastasis and 5 multiple metastatis, because the toxicities associated with combining comprehensive radiotherapy with ICI should first be established and optimized in patients with limited disease burdens. Attempting this aggressive approach in patients with high disease burdens (and correspondingly larger volumes of tumour material to be irradiated) could lead to unacceptable toxicities and subsequently to a premature aversion to combining these treatments.

Toxicity is a concern for synergy of PD-1 inhibitor and concurrent chemoradiotherapy. In our study, there was a 65% incidence of G3-4 TRAE, but the main adverse reactions were associated with chemotherapy. It was obviously lower than that in KEYNOTE 048 (72.7% vs 85%) [5]. The incidence of pneumonia of G1-4 was 27.5% in the study, only one patient had G3 radiation-induced pneumonia (2.5%), and no G4-5 pneumonia occured. Several retrospective studies have also confirmed the safety, and the incidence of pneumonia in patients who had received targeted radiotherapy of lung in combination with immune checkpoint inhibitors found no statistically significant differences [42–44]. In the PACIFIC study, all patients received a 54–66 GY dose of pulmonary radiotherapy before using durvalumab, and the incidences of pneumonia in all grades of patients using durvalumab and patients using placebo were 34% and 25%, the G3 or 4 of pneumonia were 4.4% and 3.8%, respectively [45].

Many studies have focused on the predictive biomarkers for the response of PD-L1 inhibitor, such as tumor mutational burden (TMB), PD-1 expression and Combined Positive Score (CPS) [46, 47]. Meta-analysis showed that HNSCC patients expressing PD-L1 may have a better tumor response and PFS when receiving PD-1 or PD-L1 inhibitor monotherapy, PD-L1 positive patients with ≥ 1% expression had a weighted mPFS of 3.34 months (range 1.9–13.4) [48]. The ORR and OS were higher among patients with PD-L1 positive expression [5]. A randomized Phase II Trial report the impact of PD-L1 expression in anti-PD1 immunotherapy with SBRT for metastatic HNSCC, PD-L1 positive patients was associated with higher ORR rates and better OS [26]. Our study also showed that ORR and PFS were higher among patients with PD-L1 positive status. However, due to the limited sample size, these differences were not statistically significant. Further studies and biomarkers should be used to predict the prognosis following treatment with immune checkpoint inhibitors.

Our study was a retrospective study, and it still has some limitations. The PD-1 inhibitors was not the same, and the sample size was not large enough, so we need to conduct a prospective study to reduce the bias and further confirm the results. Secondly, the optimal fractionation and total dose were not defined, and more preclinical trials should be well-designed to explore this.

## Conclusions

The combination of concurrent PD-1 inhibitor treatment with chemoradiotherapy shows promise as a treatment strategy for the R/M HNSCC patients. Compared with other studies, it could dramatically improve ORR, and prolong both PFS and OS with tolerable toxicity. It could represent a novel treatment paradigm for R/M HNSCC, and more researches should be conducted.

## Data Availability

Supporting data associated with this study are available from the corresponding author by request.
